# Daily Brief Heat Therapy Reduces Seizures in A350V IQSEC2 Mice and Is Associated with Correction of AMPA Receptor-Mediated Synaptic Dysfunction

**DOI:** 10.3390/ijms24043924

**Published:** 2023-02-15

**Authors:** Reem Jada, Veronika Borisov, Eliezer Laury, Shmuel Halpert, Nina S. Levy, Shlomo Wagner, Shai Netser, Randall Walikonis, Ido Carmi, Shai Berlin, Andrew P. Levy

**Affiliations:** 1 Technion Faculty of Medicine, Technion Israel Institute of Technology, Haifa 3200003, Israel; 2 Sagol Department of Neurobiology, Faculty of Natural Sciences, University of Haifa, Haifa 3498838, Israel; 3 Department of Physiology, University of Connecticutt, Storrs, CT 06269, USA

**Keywords:** fever, heat therapy, IQSEC2, AMPA receptor, Arf6-GTP

## Abstract

Purposeful induction of fever for healing, including the treatment of epilepsy, was used over 2000 years ago by Hippocrates. More recently, fever has been demonstrated to rescue behavioral abnormalities in children with autism. However, the mechanism of fever benefit has remained elusive due in large part to the lack of appropriate human disease models recapitulating the fever effect. Pathological mutations in the IQSEC2 gene are frequently seen in children presenting with intellectual disability, autism and epilepsy. We recently described a murine A350V IQSEC2 disease model, which recapitulates important aspects of the human A350V IQSEC2 disease phenotype and the favorable response to a prolonged and sustained rise in body core temperature in a child with the mutation. Our goal has been to use this system to understand the mechanism of fever benefit and then develop drugs that can mimic this effect and reduce IQSEC2-associated morbidity. In this study, we first demonstrate a reduction in seizures in the mouse model following brief periods of heat therapy, similar to what was observed in a child with the mutation. We then show that brief heat therapy is associated with the correction of synaptic dysfunction in neuronal cultures of A350V mice, likely mediated by Arf6-GTP.

## 1. Introduction

Mutations in the IQSEC2 gene account for 2–5% of children presenting with severe intellectual disability, autism spectrum disorder and epilepsy [1,2,3]. Children with IQSEC2 mutations are minimally verbal, have an IQ well below 50, and require constant adult care. In recent years, our laboratory has worked closely with a group of parents of IQSEC2 children to understand features of the natural history of the disorder that may provide insights into treatment. One such insight, initially made in a child with an A350V IQSEC2 mutation, was that fever was associated with a marked clinical improvement manifested as an abatement in seizures and an improvement in social interactions [4]. Moreover, purposefully raising the body temperature of the child with the A350V IQSEC2 mutation, by having the child sit in a Jacuzzi set at 39 °C twice a day for 15 min, resulted in a dramatic reduction in epileptic seizures and reduced epileptiform activity on EEG [4]. This association between fever and clinical improvement, and a reduction in seizures from purposefully raising the body temperature, has been observed in several children with different IQSEC2 mutations [5].

From antiquity to the present day, medical practitioners have investigated whether purposefully mimicking fever by raising the body temperature, either by the injection of pyrogens or by the use of a hot bath, could provide clinical benefit. Hippocrates found that fever had a beneficial effect on epilepsy [6,7]. More recently, fever has been demonstrated to rescue social behavioral deficits in some children with autism [8,9]. However, the mechanism of fever benefit has remained elusive due in large part to the lack of appropriate human disease models that can reproduce the fever association. We have recently reported on the development of human IQSEC2 disease models [10,11] that recapitulate important aspects of the human IQSEC2 disease phenotype [10,12,13,14]. CRISPR-generated A350V IQSEC2 mice develop fatal epileptic seizures between post-natal (PN) days 15–20 [14] with the surviving mice displaying profound behavioral abnormalities as adults [12]. We have further shown that seizures were dramatically reduced when we continuously housed the A350V mice at an ambient temperature of 37 °C during the PN 15–20 period; a period during which the mice exhibit seizures [14,15]. Moreover, when the A350V mice which had been treated with 5 days of continuous heat therapy as pups reached adulthood, they displayed normalized behavioral interactions [15].

We therefore sought to employ this mouse model to better understand the mechanism behind the beneficial effects of fever to then develop drugs that can mimic this effect and reduce IQSEC2-associated morbidity in man, and perhaps, extend the benefits of heat therapy towards other disorders. In the present work, we tested whether daily acute and intense exposures to heat would recapitulate the benefits of prolonged continuous heat therapy, as previously described, and examine the correction of synaptic mechanisms by this treatment.

## 2. Results

### 2.1. Measurement of Body Core Temperature in Mice

We measured the core temperature of sentinel mice with the IPTT-300 mini-transponder temperature probe from BMDS [16]. The IPTT device was implanted percutaneously with a syringe on PN day 14 and has been previously demonstrated to tightly correlate with the body core temperature measured using esophageal or rectal probes [16]. Mice were routinely housed in our SPF facility at a temperature of 22–23 °C. In order to raise the body core temperature for heat therapy treatments we transferred the mice beginning on PN day 15 to a neonatal temperature controlled incubator (Drager model 8000IC, Lubeck, Germany). At the end of the heat treatment, the mice were returned to a room with an ambient temperature of 22–23 °C.

**Figure 1:**
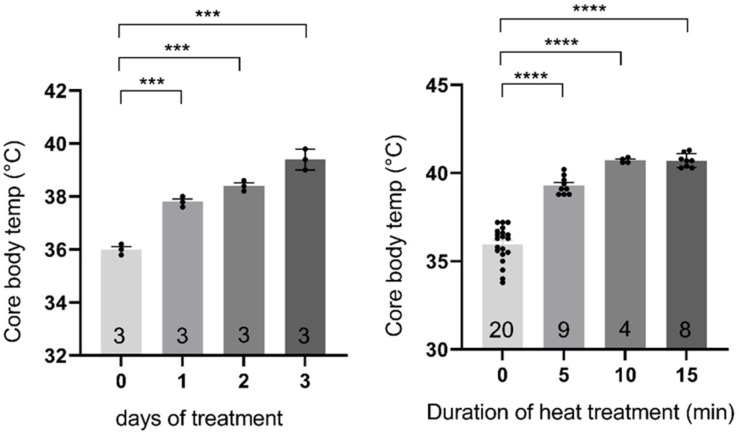
Measurement of body core temperature during heat therapy. Core body temperature during continuous exposure of mice to ambient 37 °C (**left**) or ambient 39 °C (**right**). Core body temperature was measured using the IPTT 3000 (BDMS) temperature probe inserted on PN 14 (number of mice for which temperature was measured at any time point indicated on histogram, ***, *p* < 0.0005; ****, *p* < 0.0001). A duration of 15 min of heat therapy between 15 and 20 days after birth provides protection against seizures and permits the development of contextually appropriate social vocalizations in adult A350V mice.

When the mice were housed continuously at 37 °C, we observed a gradual rise in the body core temperature over several days (Figure 1 left panel), conditions which we had previously reported provided benefit against seizures [15]. In this study we sought to determine if a short more intense exposure to heat therapy might provide benefits similar to continuous exposure at 37 °C. Previous work by Pritchard and others had established that adult mice could tolerate exposure to 39 °C for up to six hours [16]. After 15 min of exposure to 39 °C, we found that the average core temperature of PN 15 day A350V mice was approximately 40 °C with no acute mortality over the next 24 h (Figure 1 right panel).

We assessed the effect of a 15 min daily exposure of PN day 15–19 mice to an ambient temperature of 39 °C and found that this reduced lethal seizures (23% (7/31)) compared to mice which did not receive heat therapy 45% (48/108) *p* < 0.03. We did not observe any benefit from heat therapy on lethal seizures if the therapy was initiated at a younger age (d13) [mortality rate 6/15 (40%), p NS, compared to mice who did not receive treatment] or when two heat treatments spaced 6 h apart were used between PN 15–19 [mortality rate 9/30 (30%); p NS, compared to mice who did not receive treatment].

**Figure 2:**
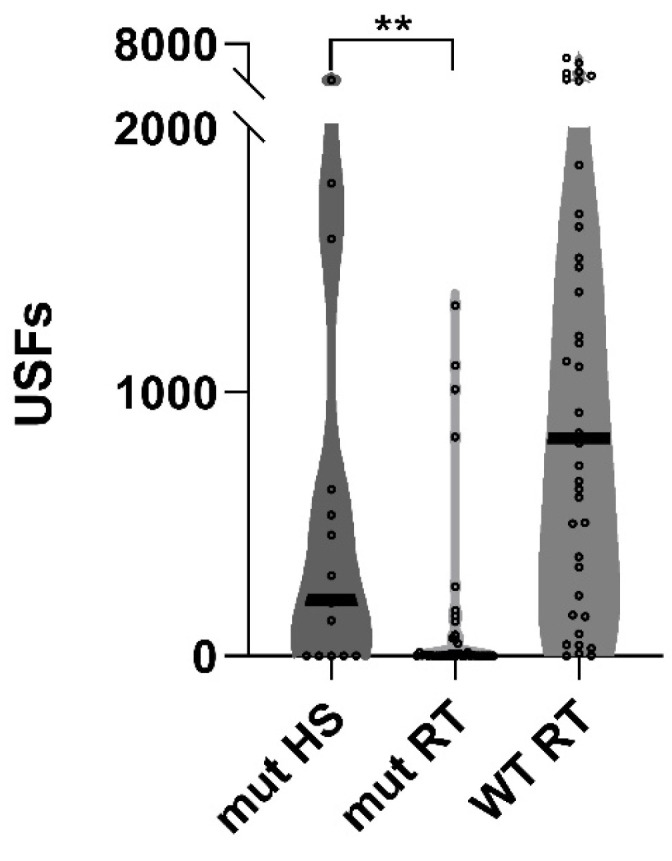
Vocalizations are increased in adult A350V IQSEC2 mice exposed to brief daily heat therapy as pups. Violin plot of ultrasonic vocalizations (USFs) in response to a female stimulus mouse were recorded from male wild-type IQSEC2 (n = 38) and male A350V IQSEC2 mutant mice (40 A350V mice no heat treatment (RT) and 15 A350V mice with heat treatment (HS)). Values for each mouse shown with small circles with thick line indicating median of group. Median was significantly greater in A350V mice undergoing heat treatment as pups as compared to A350 mice who were not treated with heat. ANOVA comparing all three groups *p* < 0.0001; specific pairwise comparison using Student’s *t*-test ** *p* = 0.01 comparing A350V ± heat shock (median number of USFs of 214 (n = 15) treated with heat therapy and 0.5 (n = 40) without heat therapy).

We have previously reported that vocalizations towards a female mouse are essentially absent in adult male A350V mice [12] and that housing A350V mice continuously at an ambient temperature of 37 °C between 15 and 20 days after birth allowed them to develop these vocalizations when they reached adulthood (8 weeks of age) [15]. We sought to determine whether short daily exposure to heat therapy at an ambient temperature of 39 °C between 15 and 19 days after birth could provide a similar benefit on vocalizations. We found that when those A350V mice who had 15 min daily treatments at 39 °C as PN 15–19-day-old pups reached adulthood (8 weeks of age) they demonstrated a significant increase in vocalizations as compared to A350V pups that had never had heat therapy (Figure 2).

### 2.2. Reproduction of the Benefit of Heat Therapy on Seizure Protection Using a Chemical Inducer of the Heat Shock Response and Loss of Benefit from Heat Therapy Using a HSF-1 Inhibitor

Celastrol [17], an inducer of the heat shock response, administered i.p. at 1 mg/kg/d from PN day 15–19 to A350V mice reduced the incidence of lethal seizures (19%; 4/21) as compared to untreated mice (45%; 48/108) (*p* < 0.03) [18]. Triptolide [19], which blocks the activation of the heat shock response, administered at 0.6 mg/kg to 15-day-old A350V mice prior to entering the 37 °C chamber, abrogated the protective effect of the heat treatment (mortality rate from seizures between d15–20 of 42% (3/7) in A350V mice treated with triptolide and heat treatment vs. 2% (1/41) in A350V mice not treated with triptolide and who received continuous heat treatment at 37 °C, *p* < 0.0004) [18]. No harm or death was observed with the same dose of triptolide administered to 14 wild-type IQSEC2 littermates treated with heat therapy. Taken together, these studies suggest that the heat shock response is important for the protective effect of heat therapy on seizures.

### 2.3. Heat Shock Decreases Arf6-GTP

**Figure 3:**
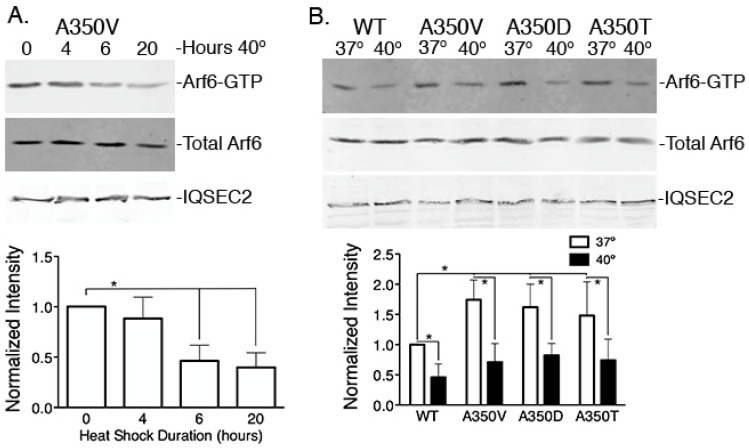
Heat shock reduces Arf6-GTP. Representative Western blot of Arf6-GTP in 293T cells transfected with (**A**) A350V IQSEC2 and cultured at 37 °C and indicated amounts of time at 40 °C or (**B**) transfected with WT and A350 mutants and cultured at either 37 °C vs. 40 °C (20 h). There were no differences in total Arf6 in any of the lines or with treatment at 40 °C. Arf6-GTP from the cells exposed to heat shock was normalized to cells transfected with A350V (**A**) or WT IQSEC2 (**B**) and incubated at 37 °C (n = 6/condition) * *p* < 0.01 ANOVA with Tukey–Kramer post hoc test.

We sought to understand the mechanism by which heat shock protects against seizures in A350V mice. The major function of IQSEC2 is to promote exchange of GDP for GTP on Arf6 [20], thereby activating Arf6 for its role in downregulating neuronal surface AMPA receptors (AMPAR) which are major regulators of cellular excitability [21,22]. We have previously shown that the A350V IQSEC2 missense mutation causes constitutive activation of its GEF function resulting in an elevation of Arf6-GTP [10]. We have extended this observation of constitutive elevation of Arf6-GTP to two additional human mutations at IQSEC2 residue 350 (A350T and A350D). We assessed the effect of brief heat shock on Arf6-GTP in 293T cells transfected with the A350V mutant IQSEC2. Arf6-GTP was compared between cells at 37 °C and those exposed to 40 °C for 4–20 h (Figure 3A). We found that heat treatment of cells transfected with A350V resulted in an approximately 50–60% reduction in Arf6-GTP (Figure 3) with no change in total Arf6. This effect was seen not only for the A350V mutant but also for other missense mutants (A350D/A350T) and wild-type IQSEC2 (Figure 3B).

### 2.4. Abnormalities in sAMPAR Attributed to Elevated Arf6-GTP Are Corrected by Heat Shock

**Figure 4:**
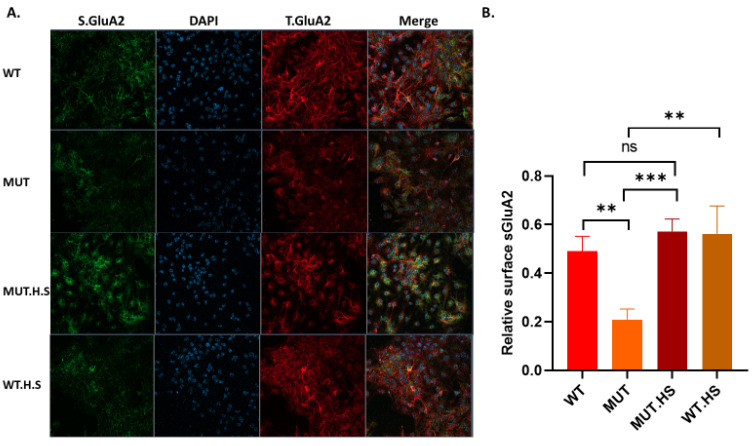
Surface GluA2 AMPA receptor in neonatal hippocampal neurons is decreased in A350V neurons and is increased after heat treatment. (**A**) Representative images (20× magnification) of total (T.GluA2) and surface (S.GluA2) GluA2 AMPA receptor in wild-type (WT) and mutant (A350V) IQSEC2 hippocampal neurons. Neurons treated with heat (40 °C) for 1 h are labelled as MUT.H.S or WT.H.S. (**B**) Quantification of relative surface GluA2 expression (ratio: S.GluA2/T.GluA2). Data are reported as the mean ± SEM obtained from at least 4 neuronal culture preparations for each of the 4 groups. ** *p* < 0.01, *** *p* < 0.001, n.s. *p* > 0.05.

Arf6-GTP has a direct effect on surface expression of AMPA receptors (sAMPAR). We have previously shown that the increase in Arf6-GTP due to the A350V mutation is associated with a reduction in sAMPAR in human A350V iPSC-derived hippocampal neurons compared to CRISPR corrected controls [10,11]. We found a similar reduction in sAMPAR in A350V IQSEC2 murine hippocampal neuronal cultures compared to wild-type IQSEC2 murine hippocampal neuronal cultures (Figure 4A). We found that heat shock (40 °C for 1 h) of A350V IQSEC2 murine hippocampal neurons increased sAMPAR (Figure 4B) to levels that were seen in wild-type IQSEC2 neurons.

### 2.5. Abnormalities in AMPAR-Mediated Spontaneous Synaptic Transmission Are Corrected by Heat Shock

**Figure 5:**
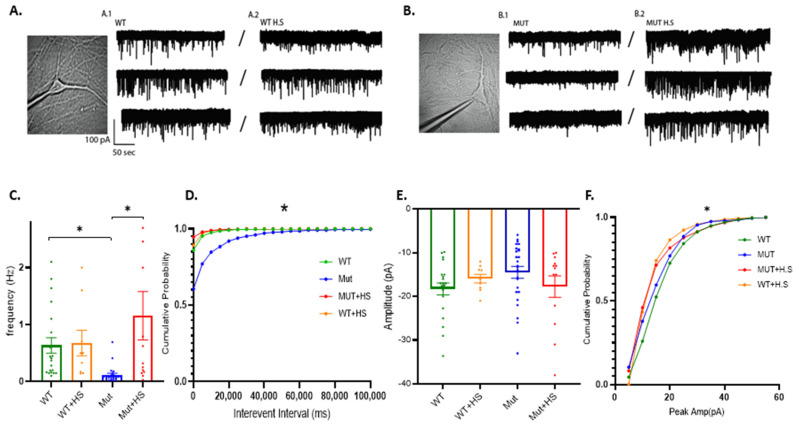
Heat treatment normalizes synaptic activity in mutant A350V neonatal hippocampal neurons. Recordings were obtained for 21 wild-type and 25 mutant neurons without heat treatment and compared to 10 wild-type and 13 mutant neurons after heat treatment (40 °C for 1 h). (**A**) Representative trace of mEPSC_AMPAR_ of a wild-type neuron before (**A.1**) and after (**A.2**) heat shock treatment at 40 °C for one hour. (**B**) Representative trace of mEPSC_AMPAR_ of an IQSEC2 A350V mutant neuron before (**B.1**) and after (**B.2**) heat shock treatment at 40 °C for one hour. (**C**) There was a significant reduction in the mEPSC_AMPAR_ mean frequency in MUT compared to WT neurons (*, *p* = 0.0002). Heat treatment resulted in a significant increase in the mEPSC_AMPAR_ mean frequency in MUT neurons (*, *p* = 0.001) which was not significantly different from that seen in WT neurons with or without heat treatment. (**D**) Cumulative probability of mEPSC_AMPAR_ frequency (*, *p* = 0.0001 by ANOVA for all 4 groups). (**E**) There was a small but non-significant difference in the mean mEPSC_AMPAR_ amplitude between MUT and WT neurons (*p* = 0.06). (**F**) Cumulative probability of mEPSC_AMPAR_ amplitude (*, *p* = 0.05 by ANOVA for all 4 groups).

We have previously demonstrated abnormal spontaneous synaptic transmission in human A350V IPSC derived hippocampal neurons compared to CRISPR corrected controls [11]. Here, in order to try to understand the synaptic mechanisms behind the benefits of heat therapy, we explored AMPAR-dependent miniature excitatory post-synaptic currents (mEPSC_AMPAR_) in cultured murine hippocampal neurons with and without heat treatment. We patched DIV 7–9 hippocampal neurons obtained from PN 0–1 A350V (MUT) or wild-type (WT) IQSEC2 pups and find that MUT neurons exhibit markedly reduced mEPSC_AMPAR_ frequency and a slight non-significant reduction in amplitude compared to WT neurons (Figure 5A–E (green and blue)). Exposure of MUT neurons to 40 °C for one hour corrected the major deficits in mEPSC_AMPAR_ frequency (Figure 5A–E (orange and red)). There were no significant differences by ANOVA in the resting potential, resistance or capacitance of neurons patched between groups (Supplemental Table S1).

## 3. Discussion

This study was motivated by the desire to understand how fever therapy may provide clinical benefit for at least some IQSEC2 mutations and whether this could be exploited for treating patients. Our finding that brief periods of heat treatment or treatment with a drug such as Celastrol, that mimics heat therapy, reduces seizures in the A350V IQSEC2 model suggests important clinical implications. IQSEC2 disease is a disorder in which seizures are generally pharmacoresistant and the heavy seizure burden in children with IQSEC2 disease plays an important role in the development of their epileptic encephalopathy [23,24]. The benefit on seizure reduction in the A350V murine model with daily 15-min treatments mirrors what we have previously reported in a child with the A350V mutation undergoing 15-min Jacuzzi treatments [4]. Moreover, we have identified other children with different IQSEC2 mutations whose seizures decrease with fever and it appears that some of these children have a reduction in seizure frequency with daily heat therapy [5].

Mechanistically, we sought to understand the molecular basis for benefit provided by brief periods of heat therapy. We therefore focused our attention on effectors of IQSEC2, specifically on the increase in activity of Arf6 (Arf6-GTP) [10]. We have previously shown that the A350V mutation leads to an increase in Arf6-GTP which is known to decrease neuronal surface AMPAR [21,22] and that this downregulation of surface AMPAR is observed both in iPSC patient derived A350V hippocampal neurons and in the murine A350V model [10,11]. However, how would a reduction in AMPAR receptors and AMPAR-mediated synaptic transmission result in seizures? The most plausible explanation is the concomitant reduction in AMPAR in inhibitory neurons which leads to a dysregulation of the excitation-inhibition (E/I) balance. In support, recent work by Sah et al. has shown the important role of IQSEC2-mediated control of excitability and AMPAR on inhibitory interneurons in the hippocampus [25]. In addition, the effect of the A350V mutation appears to primarily affect the GluA2 subunit, as compared to other AMPAR family members, and this type of imbalance has been implicated in seizure propagation in the CDKL5 model [26]. Lastly, induction of seizures by reduced surface expression or activity of excitatory (ionotropic) glutamate channels is also observed in diseases involving analogous glutamate receptors. For instance, Kellner et al. have only recently reported two de novo mutations within the N-methyl-D-aspartate receptor (NMDAR) that cause a dramatic drop in affinity for glutamate [27]. These mutations render these channels virtually inactive at the synapse; however, patients exhibit seizures likely as a result of the same E/I imbalance.

The pathophysiological pathways underlying disease in individuals carrying different IQSEC2 mutations (knockout and missense) appear to converge at the level of increased amounts of activated Arf6 (bound to GTP) relative to inactivated Arf6 (bound to GDP) [28]. We propose that constitutively elevated Arf6-GTP is responsible for the synaptic dysfunction resulting in seizures and behavioral and cognitive abnormalities observed with IQSEC2 mutations and that heat therapy provides benefit by reducing Arf6-GTP levels. This hypothesis may be tested by using mutants of Arf6 that effectively clamp Arf6 in a GTP or GDP bound state [29]. The cycling of Arf6 between its inactive GDP and active GTP bound form is dependent on the activity of a family of GEF (GTP exchange factors) such as IQSEC2 and GAP (GTPase activating factors). Heat therapy may result in a reduction in Arf6-GTP either thru a reduction in GEF activity or an increase in GAP activity for Arf6 or even by auto-activation of the intrinsic labile GTPase activity of Arf6. This question may be approached using a real-time NMR assay to monitor Arf6-GTP/GDP cycling in cell and tissue extracts via nucleotide-dependent structural changes in isotopically labeled Arf6 [30]. Identification of a downregulation of Arf6-GTP as the key step by which fever provides benefit may be further verified in our murine models using drugs [31,32,33,34] which can decrease levels of Arf6-GTP, thereby providing a precision medicine approach to treat IQSEC2 disease.

Another potential pathway by which heat shock provides therapeutic benefit may be thru SIRT3. SIRT3 has recently been demonstrated to provide protection from heat stress. Specifically, SIRT3 overexpression was found to provide mitochondrial protection and decreased oxidative stress which normally occurs in response to heat stress [35]. Moreover, pharmacological activation of SIRT3 with honokiol was found to promote neuronal survival and differentiation, suggesting a possible novel therapeutic [36].

We have observed that heat treatment markedly increases the frequency of mEPSPs and to a lesser extent the amplitude of the mEPSPs. Traditionally, this would indicate that the effect is predominately presynaptic. However, it is also possible that heat therapy serves to upregulate AMPAR at synapses which that are lacking in sAMPAR (i.e., silent synapses) [37,38]. This latter hypothesis is consistent with the marked increase in sAMPAR at the postsynaptic density with heat treatment, and is supported by reports showing reduction in miniature EPSCs that result from loss-of-function mutations in the other glutamate receptors [39].

This study provides the framework to understand how fever may provide clinical benefit and a model system that may allow for the translational application of heat therapy to the clinic for diseases that extend far beyond the A350V IQSEC2 mutation. An emerging paradigm in precision medicine is that apparently different diseases which share pathophysiological pathways, regardless of their etiology, may allow a therapy from one of the diseases to be repurposed for the other. One example of this approach may be towards the rescue of the defect in synaptic transmission seen in Alzheimer’s disease (AD). Arf6 activation, similar to what occurs in A350V, is increased in AD and this plays a direct role on the cleavage of the amyloid precursor protein and generation of amyloid plaques [40,41]. These plaques in turn have been demonstrated to result in marked reduction in sAMPAR and synaptic dysfunction manifested as decreased spontaneous EPSCs [42]. Two recent studies have shown a positive effect of prolonged heat therapy on AD. First, in a population based longitudinal study of 2315 middle aged men followed for a median of 20 years Laukkanen et al. [43] found that moderate to high frequency heat treatments in a sauna (4–7 times a week) were associated with a 65% reduction in the incidence of AD (HR 0.35 (95% CI 0.14–0.90). Second, in a prospective cohort study of 13,994 men and women aged 30–69 followed over a 20 year period the development of dementia between individuals receiving heat treatment in a sauna 9–12 times a month was reduced over 50% as compared to individuals not getting heat treatments in a sauna or with a frequency of sauna treatments of less that four times a month (HR 0.47 (95% CI 0.25–0.88) [44]. Thus, it would be of interest to examine if therapies targeting Arf6, or drugs such as celastrol that can mimic heat therapy, might show benefit in AD.

## 4. Materials and Methods

### 4.1. Animals

The generation of A350V IQSEC2 mice in a C57BL6/J background has previously been described [10]. All mice used in this study were generated from a cross between a female heterozygous for the A350V mutation and a wild-type IQSEC2 male resulting in half of the offspring having the mutation (hemizygous in males) and half being wild-type for IQSEC2. Only male mice were used for experiments to avoid the confounding effects of X-inactivation in females. Mice were generally housed in a germ free facility with free access to water and food at 22 °C. All animals were kept on a 12-h light\12-h dark cycle, light on at 9 p.m., with ad libitum access to food and water. Behavioral experiments took place during the dark phase under dim red light. Mice were genotyped when they were 12–13 days old by polymerase chain reaction (PCR) as previously described [10]. Use of these mice in the studies described in this study was approved by the Institutional Animal Care and Use Committee of the Technion Israel Institute of Technology (protocol #IL-114-09-20).

### 4.2. Assessment of Male Female Ultrasonic Vocalizations

Ultrasonic vocalizations were recorded using a condenser ultrasound microphone (Polaroid/CMPA, Avisoft Bioacoustics, Glienicke/Nordbahn, Germany). The microphone was connected to an ultrasound recording interface (UltrasoundGate 116Hme, Avisoft Bioacoustics, Glienicke/Nordbahn, Germany), which was plugged into a computer equipped with the recording software Avisoft Recorder USG (sampling frequency: 250 kHz; FFT-length 1024 points; 16-bit format). Vocalizations were recorded in 8–10-week-old male wild-type or A350V mice during a 5 min interaction with a female C57Bl/6 stimulus, following a 15 min of habituation to the arena [14]. Ultrasonic vocalizations were analyzed using our TrackUSF custom-made software version 1.0 (Wagner lab, Haifa, Israe) [45]. Data for vocalizations are reported as ultrasonic fragments (USFs), the number of which is directly proportional to the total time of ultrasonic vocalizations.

### 4.3. Chemicals Used in This Study

Celastrol (Fermentek Ltd., Jerusalem, Israel) was dissolved in 100% DMSO at a concentration of 10 mg/mL and stored at −20 °C for up to one week prior to dilution in PBS immediately prior to i.p. injection at a dose of 1 mg/kg/d. Triptolide (Sigma, Rehovot, Israel) was dissolved in 100% DMSO and stored at −20 °C prior to dilution in PBS immediately prior to ip injection at a dose of 0.6 mg/kg. Tetrodotoxin (TTX), 2-(3-Carboxypropyl)-3-amino-6-(4 methoxyphenyl) pyridazinium bromide Gabazine (GBZ), and DL-2-amino-5-phosphonovaleric acid (APV) were obtained from Alomone labs (Jerusalem, Israel).

### 4.4. Assessment of Arf6-GTP

We measured Arf6-GTP in HEK293T cells transiently transfected with IQSEC2 carrying IQ domain mutations (A350V, A350T, A350D, R359C, or E849K IQSEC2) cloned into pCAGGS and HA-tagged Arf6 in pXS [10]. The relative amount of ARF6 bound to GTP as compared to total ARF6 was assessed using a GGA-3 pulldown assay and Western blot as previously described [46]. Bands were visualized using a LiCor Odyssey imaging system and quantified with Image Studio Lite. Each band was normalized to the untreated sham-transfected control.

### 4.5. Preparation of Neonatal Hippocampal Neuronal Cultures

A350V and wild-type IQSEC2 neurons were prepared as previously reported with some modifications [47]. Hippocampi were extracted from mouse neonates (P0-1), dissociated in HBSS medium (Ca^+2^- and Mg^2+^-free) and then transferred to 24-well plates containing poly-D-lysine-covered sterilized glass coverslips and MEM (Gibco)-based growth medium and cultured in a 37 °C 5% CO_2_ incubator. After 5 days in vitro (DIV), the neuronal culture medium was supplemented with 4 mM cytosine arabinoside (ARA-C) for suppression of glial cell proliferation. Subsequently, half of the culture media was replaced every other day with fresh neuronal growth media. At 7–9 days in vitro (DIV) patch clamp recording/immunocytochemistry staining was performed. For heat treatment, cultures at DIV 7 were transferred to a CO_2_ incubator set at 40 °C for one hour followed by patch clamp recording/immunocytochemistry analysis immediately thereafter.

### 4.6. Immunohistochemistry of Murine Hippocampal Neuronal Cultures for Surface AMPA Receptor

Neurons were fixed with 4% PFA in PBS followed by three washes with PBS and blocking with 5% normal goat serum in PBS for one hour at room temperature. To label neuronal surface GluA2 AMPAR, coverslips were incubated at 4 °C overnight in PBS containing 3% normal goat serum and mouse monoclonal extracellular anti-N-terminal GluA2 IgG (Synaptic System, Gottingen, Germany, cat# 182111). After four washes with PBS, coverslips were incubated with secondary goat-anti mouse ALEXA FLOUR 488 (Jackson Immuno Research, WestGrove, PA, USA, cat # 115-545-166) for one hour at room temperature. After four washes with PBS, cells were permeabilized and intracellular GluA2 labeled with 0.3% Triton X-100, 3% normal goat serum in PBS and 1% rabbit polyclonal intracellular anti-C-terminal GluA2 antibody (Synaptic System, cat # 182103) for 16 h at 4 °C. After four washes with PBS, the coverslips were then incubated in PBS containing ALEXA FLOUR 647 goat anti-rabbit IgG secondary antibody (Jackson Immuno Research, cat # 111-165-045) for one hour at room temperature followed by staining with DAPI for 5 min. After four washes with PBS, images were acquired using a Zeiss LSM laser-scanning confocal microscope. Images for all conditions were analyzed by using identical acquisition parameters. The fluorescence intensities of labelled surface (AFlour 488) and total (AFlour 647) staining were measured using ZEN software version 3.5.093 (Zeiss, Oberkochen, Germany). Surface expression was determined by the ration of surface signal normalized to the total signal. Data are presented as the mean ± SEM with groups compared by ANOVA followed by specific pairwise comparisons using Student’s *t*-test with a *p* < 0.05 being considered statistically significant.

### 4.7. Patch Clamp Recordings

Recordings were performed using commercial amplifier (Warner Instruments, Holliston, MA, USA) and Digitizer (Digidata-1550B; Molecular Devices, San Jose, CA, USA), controlled by pClamp10 software version 10.7 (Molecular Devices, San Jose, CA, USA). Electrodes consisted of pulled glass capillaries (micropipette puller model P-97, Sutter Instruments, Novato, CA, USA) with chlorinated silver wire, with a resistance of 8–12 MΩ when filled with intracellular solutions (in mmol/L): 135 K-gluconate, 10 NaCl, 10 HEPES, 2 MgCl_2_, 2 Mg ATP and 1 EGTA, adjusted to pH 7.3 with KOH. Coverslips with cells were perfused with extracellular bath solution in (mmol/L): 138 NaCl, 1.5 KCl, 1.2 MgCl_2_, 10 d-glucose, 2.5 CaCl_2_, 5 HEPES adjusted to pH 7.4 with NaOH [47].

### 4.8. AMPA Miniature EPSCs Recordings in Neonatal Hippocampal Neurons

Spontaneous miniature excitatory postsynaptic currents (mEPSCs) were recorded in the whole-cell voltage-clamp mode. Neurons were held at −70 mV and recordings were obtained in the presence of the sodium channel blocker TTX (1–2 μM), the GABA antagonist,—GBZ (10 μM) and the NMDA antagonist APV (100 μM) in the extracellular solution. For heat treatment, cells were incubated at 40 °C for 1 h and then recorded immediately. Data were acquired by Axon MultiClamp model 700B and Axon Digidata 1440 acquisition systems (Molecular Devices, San Jose, CA, USA) at a at a sampling rate of 20 kHz. mEPSCs were analyzed offline by Clampfit software version 10.6.0.13 (Molecular Devices, San Jose, CA, USA). Statistical significance between groups was assessed by one-way ANOVA followed by specific pairwise comparisons using Student’s *t*-test, with *p* values of <0.05 considered statistically significant.

## Data Availability

All data in this manuscript can be obtained by contacting the corresponding author.

## Supplementary Materials

The following supporting information can be downloaded at: https://www.mdpi.com/article/10.3390/ijms24043924/s1.
