# Supplementary material for: Daily Brief Heat Therapy Reduces Seizures in A350V IQSEC2 Mice and Is Associated with Correction of AMPA Receptor-Mediated Synaptic Dysfunction
Source: Int J Mol Sci. 2023 Feb 15;24(4):3924. doi: 10.3390/ijms24043924 (PMC9965438; doi:10.3390/ijms24043924)
Supplement: Supplementary file 1 [file ijms-24-03924-s001.zip › ijms-2227110-supplementary.pdf]

Table S1. Summary of membrane properties of WT and MUT neurons with and without heat shock (HS)

	Rm (mΩ)	Cm (pF)	Vm (mV)
**WT**	498.19±71.07	31.95±2.7	-40.26±2.01
**WT.HS**	411±43.14	41.78±2.03	-43.56±3.27
**MUT**	656.20±42.45	27.85±1.53	-38.92±1.97
**MUT.HS**	451.85±39.75	39.46±3.47	-47.69±2.74

Rm, resistance; Cm, capacitance; Vm, resting membrane potential. N (neurons) for each group as defined in Figure 5 of the manuscript. There were no significant differences across the groups for any parameter by ANOVA.
